# Hepatic Steatosis Contributes to the Development of Muscle Atrophy *via* Inter-Organ Crosstalk

**DOI:** 10.3389/fendo.2021.733625

**Published:** 2021-10-11

**Authors:** Kenneth Pasmans, Michiel E. Adriaens, Peter Olinga, Ramon Langen, Sander S. Rensen, Frank G. Schaap, Steven W. M. Olde Damink, Florian Caiment, Luc J. C. van Loon, Ellen E. Blaak, Ruth C. R. Meex

**Affiliations:** ^1^ Department of Human Biology, School of Nutrition and Translational Research in Metabolism (NUTRIM), Maastricht University, Maastricht, Netherlands; ^2^ Maastricht Centre for Systems Biology, Maastricht University, Maastricht, Netherlands; ^3^ Department of Pharmaceutical Technology and Biopharmacy, Groningen Research Institute of Pharmacy, University of Groningen, Groningen, Netherlands; ^4^ Department of Respiratory Medicine, School of Nutrition and Translational Research in Metabolism (NUTRIM), Maastricht University, Maastricht, Netherlands; ^5^ Department of Surgery, School of Nutrition and Translational Research in Metabolism (NUTRIM), Maastricht University, Maastricht, Netherlands; ^6^ Department of General, Visceral and Transplantation Surgery, RWTH University Hospital Aachen, Aachen, Germany; ^7^ Department of Toxicogenomics, School of Oncology and Developmental Biology (GROW), Maastricht University, Maastricht, Netherlands

**Keywords:** hepatic steatosis, NAFLD, inter-organ crosstalk, muscle atrophy, sarcopenia, insulin resistance, metabolism

## Abstract

Individuals with hepatic steatosis often display several metabolic abnormalities including insulin resistance and muscle atrophy. Previously, we found that hepatic steatosis results in an altered hepatokine secretion profile, thereby inducing skeletal muscle insulin resistance *via* inter-organ crosstalk. In this study, we aimed to investigate whether the altered secretion profile in the state of hepatic steatosis also induces skeletal muscle atrophy *via* effects on muscle protein turnover. To investigate this, eight-week-old male C57BL/6J mice were fed a chow (4.5% fat) or a high-fat diet (HFD; 45% fat) for 12 weeks to induce hepatic steatosis, after which the livers were excised and cut into ~200-µm slices. Slices were cultured to collect secretion products (conditioned medium; CM). Differentiated L6-GLUT4myc myotubes were incubated with chow or HFD CM to measure glucose uptake. Differentiated C2C12 myotubes were incubated with chow or HFD CM to measure protein synthesis and breakdown, and gene expression *via* RNA sequencing. Furthermore, proteomics analysis was performed in chow and HFD CM. It was found that HFD CM caused insulin resistance in L6-GLUT4myc myotubes compared with chow CM, as indicated by a blunted insulin-stimulated increase in glucose uptake. Furthermore, protein breakdown was increased in C2C12 cells incubated with HFD CM, while there was no effect on protein synthesis. RNA profiling of C2C12 cells indicated that 197 genes were differentially expressed after incubation with HFD CM, compared with chow CM, and pathway analysis showed that pathways related to anatomical structure and function were enriched. Proteomics analysis of the CM showed that 32 proteins were differentially expressed in HFD CM compared with chow CM. Pathway enrichment analysis indicated that these proteins had important functions with respect to insulin-like growth factor transport and uptake, and affect post-translational processes, including protein folding, protein secretion and protein phosphorylation. In conclusion, the results of this study support the hypothesis that secretion products from the liver contribute to the development of muscle atrophy in individuals with hepatic steatosis.

## Introduction

Non-alcoholic fatty liver disease (NAFLD) is the most common form of chronic liver disease and encompasses a histological spectrum of liver diseases. It starts with hepatic steatosis and can progress to non-alcoholic steatohepatitis (NASH), cirrhosis, and hepatocellular cancer. Hepatic steatosis is a key feature of NAFLD and is diagnosed when more than 5% of the total liver weight consists of fat. Hepatic steatosis is highly prevalent and is found in ~25% of all adults (1, 2), in up to ~70% of adults who are overweight, and in >90% of the individuals who are morbidly obese (3–5), indicating a close link with excess body weight.

Hepatic steatosis has previously been considered benign, although this dogma has been challenged in recent years. Hepatic steatosis is linked to metabolic abnormalities, including hypertriglyceridemia, type 2 diabetes, and cardiometabolic diseases. Furthermore, the liver has been recognized as an endocrine organ that secretes hepatokines to influence metabolism locally and in distant tissues *via* inter-organ crosstalk, and we previously found that hepatic steatosis changes the protein secretion pattern of hepatocytes, resulting in insulin resistance in skeletal muscle cells (6).

In the past decade, there has been a growing interest in the relationship between NAFLD and sarcopenia (7). Sarcopenia is the loss of muscle mass and muscle strength that is inherent to aging. In healthy individuals, muscle mass decreases at an annual rate of 1% to 2% after the age of 50 (8), which means that an average male person of 80 kg with 35 kg of muscle mass would lose 350 to 700 g per year, the equivalent of 7 to 14 kg over 20 years. In individuals with NAFLD, the prevalence of sarcopenia is strongly increased and correlates with the severity of steatosis and fibrosis (9, 10). Specifically, the prevalence of sarcopenia in subjects without NAFLD, with hepatic steatosis, and with NASH was found to be 8.7%, 17.9%, and 35.0%, respectively (11). Additionally, in individuals with advanced NAFLD, NASH was associated with a 6-fold increased risk of developing sarcopenia (12), and the loss of muscle mass was associated with decreased survival, increased length of hospitalization, and increased mortality (13). Due to the cross-sectional nature of these studies, the exact relationship between NAFLD and sarcopenia in terms of cause and consequence remains obscure, however, and it is not known if sarcopenia contributes to NAFLD or vice versa. It has been suggested for the first time in two Korean studies that sarcopenia could be involved in the etiology of NAFLD (14, 15). In a large Korean cohort of 9,565 individuals, multivariate regression analysis showed that the skeletal muscle to visceral fat ratio was inversely correlated to hepatic steatosis, and it was suggested that a higher skeletal muscle mass may have a beneficial effect in preventing NAFLD (15). In the ‘Korean Sarcopenic Obesity Study’, in 452 apparently healthy adults it has been shown that individuals with low muscle mass had an increased risk of NAFLD, even after adjusting for confounding factors, including insulin resistance and inflammation (14). Since the publication of these two studies, the majority of studies have suggested that sarcopenia is a risk factor for the development of NAFLD. A recent meta-analysis including 19 studies reported that patients with sarcopenia have an increased risk of developing hepatic steatosis, as well as advanced NAFLD stages, including NASH and fibrosis (9). Importantly though, the studies in this meta-analysis also cannot differentiate between cause and consequence. It is thus possible that NAFLD leads to muscle loss, rather than the other way around. In support of this hypothesis, it has been found in patients who underwent a liver transplantation that sarcopenia did not progress, but was arrested and frequently improved (16). This was observed in the absence of confounding conditions such as recurrent allograft liver disease or post-transplant complications (16). Given our previous finding that hepatic steatosis contributes to the development of insulin resistance in skeletal muscle *via* inter-organ crosstalk (6), we hypothesized that hepatic steatosis and its related secretome contribute to the development of sarcopenia *via* effects on muscle protein metabolism.

To investigate this, male C57BL/6J mice were fed a chow or a high-fat diet (HFD) to induce hepatic steatosis, after which the livers were sliced and cultured. Secretion products were collected and transferred to differentiated myotubes for 24 h to measure muscle protein synthesis and protein breakdown rates. Interestingly, our results show that the secretion products of a fatty liver do not affect protein synthesis in differentiated myotubes, but increase muscle protein breakdown rates. In line with this, using RNA sequencing, we found that pathways related to muscle morphology and function were strongly enriched in the myotubes incubated with the HFD liver secretome. These findings support our hypothesis that hepatic steatosis contributes to the development of sarcopenia in individuals with NAFLD *via* inter-organ crosstalk.

## Materials and Methods

### Animal Studies

All experimental and surgical procedures were approved by the Animal Ethics Committee of Maastricht University (DEC-2017-002). Sixteen male C57BL/6J mice were purchased at 8 weeks of age and randomly assigned to an *ad libitum* chow diet (4.5% fat) or HFD (45% fat, ssniff^®^) for 12 weeks to induce hepatic steatosis. Mice had access to water at all times and were housed under controlled temperature (21°C) and lighting (12h:12h light-dark cycle). Body weight was measured weekly. On each experimental day, two mice were anaesthetized and the liver was perfused *via* the hepatic portal vein with University of Wisconsin (UW) solution to free the liver from blood. The liver was excised and placed in ice-cold UW solution to preserve the organ for tissue slicing. The left epididymal fat pad of the mouse was excised as an additional indicator of adiposity.

### Precision-Cut Liver Slices

Precision-cut liver slices (PCLS) were collected in accordance with a protocol by de Graaf et al., originally designed for rat livers (17), with some minor modifications. Briefly, the mouse liver was placed in a petri dish and covered with fresh, ice-cold UW solution. A 5-mm biopsy punch was used to create liver tissue cores, which were then transferred to fresh, ice-cold UW solution. Remaining liver tissue was snap-frozen in liquid nitrogen and stored for further analyses. The liver cores were placed in a Krumdieck tissue slicer, filled with ice-cold Krebs buffer. The Krebs buffer was previously oxygenated with carbogen (95% O_2_ and 5% CO_2_), pH adjusted to 7.42, and sterilized by filtration with a 0.45-µm pore filter. The Krumdieck tissue slicer was used to cut liver slices of approximately 5 mg wet weight, which corresponds to a thickness of approximately 200 µm. Only slices with a round shape, a uniform thickness, and smooth edges were selected and transferred to ice-cold UW solution. It has previously been shown that liver slices remain viable for up to 96 h (17).

### Collection of Conditioned Medium

12-wells plates containing 1.3 mL/well Williams’ Medium E + GlutaMAX, with 2.5 mg/mL D-glucose, and 50 µg/mL gentamicin were placed on a shaker at 90 RPM inside a cell incubator that was set to 37°C, 80% O_2_, 5% CO_2_. Each individual slice was transferred to a single well for a pre-incubation of approximately 2.5 h to allow the restoration of ATP content and the removal of cell debris. The slices were washed twice with warm PBS, after which they were incubated for 24 h in 1.3 mL/well EX-CELL^®^ 325 protein-free, serum-free medium. The conditioned medium (CM) was collected, centrifuged for 3 min at 4°C and 100 g, and the supernatant was taken and stored at -80°C. Fresh medium was added for a second incubation period of 24 h, followed by the same collection steps.

### Triacylglycerol Assay

Liver tissue that was left over after creation of the liver cores was snap frozen and used to measure lipid content. The lipids in the liver slices were extracted overnight in chloroform:methanol (2:1). Triacylglycerol (TAG) content was determined by enzymatic colorimetric assay (GPO-PAP reagent, Roche Diagnostics) and expressed as µmol TAG/mg liver tissue.

### Cell Culture

C2C12 and L6-GLUT4myc myoblasts were cultured in low-glucose Dulbecco’s Modified Eagle Medium (DMEM; ThermoFisher Scientific) with 10% fetal bovine serum (FBS) and 1% penicillin-streptomycin at 37°C, 21% O_2_, 5% CO_2_. To induce differentiation, cells were plated onto 12-wells (for protein synthesis and breakdown assays) or 24-wells culture plates (for glucose uptake and gene expression assays) that were coated with 2% (*v*/*v*) Growth Factor Reduced Matrigel^®^ (BD Biosciences, Bedford, MA) and incubated in high-glucose DMEM with 2% FBS and 1% penicillin-streptomycin. DMEM was replaced every other day.

### Glucose Uptake

For the glucose uptake assay, L6-GLUT4myc cells were differentiated for 5 days, washed with warm PBS, and incubated for 24 h with chow CM or HFD CM to which 2% FBS was added. CM was then aspirated and cells were washed with warm PBS. Cells were pre-incubated for 10 min in no-glucose DMEM containing 0.1% bovine serum albumin and 10 µM 2-deoxy-D-glucose either with or without 10 nM insulin. After 10 min, medium was aspirated and the cells were incubated for 10 min in the same medium either with or without insulin, and with an additional 1 µCi/mL 2-[1,2-^3^H(N)]-deoxy-D-glucose (PerkinElmer, NET328A001MC). Medium was aspirated, cells were washed three times with cold PBS, and 1 M NaOH containing 0.1% Triton X-100 was added. The cells were scraped and transferred to vials containing Ultima Gold scintillation liquid. The level of radioactivity was measured by liquid scintillation counting (PerkinElmer liquid scintillation counter). (n = 7/6 biological replicates per group and 3 technical replicates.)

### Protein Synthesis and Breakdown

To measure protein synthesis, C2C12 cells were differentiated for 5 days. They were then washed with warm PBS and incubated with CM, supplemented with 2% FBS, for 16 h. After 16 h, 0.3 mM L-phenylalanine and 0.1 µCi/mL L-[^14^C(U)]-phenylalanine (PerkinElmer, NEC284E050UC) were added, and cells were incubated for an additional 8 h. The dilution of the CM by L-phenylalanine was 5%. Medium was then aspirated, cells were washed three times with cold PBS, and incubated with 1 M perchloric acid for 1 h at 4°C. Cells were washed twice with perchloric acid and once with PBS, after which 1 M NaOH containing 0.1% sodium dodecyl sulfate was added. Cells were left overnight at 37°C and scraped the following morning. The samples were added to vials containing Ultima Gold scintillation liquid. The level of radioactivity was measured by liquid scintillation counting, and corrected for protein content. (n = 7/6 biological replicates and 4 technical replicates). For **Supplementary Figure 1**, protein synthesis was performed after 3 days of differentiation with a variety of control conditions. Protein synthesis was measured after incubation with differentiation medium (DM) in the unstimulated condition or with the addition of 100 nM insulin or 10 nM insulin-like growth factor-1 (IGF-1). Protein synthesis was also measured after incubation with CM in the absence or presence of 100 nM insulin.

For the protein breakdown assay, C2C12 cells were incubated for 24 h in DM containing 0.2 µCi/mL L-[^14^C(U)]-phenylalanine. After 24 h, the medium was aspirated and cells were washed two times with DMEM. Cells were then incubated for 2 h in DM containing 0.3 mM non-radioactive L-phenylalanine. Medium was aspirated and cells were washed again two times with DMEM. Cells were then incubated for 24 h with either chow or HFD CM containing 0.3 mM non-radioactive L-phenylalanine and 1% FBS. After 24 h, medium was collected, to which 1 M perchloric acid was added to allow precipitation on ice for 1 h. The medium was then centrifuged at 13,000 RPM for 5 min. Supernatant was taken and added to vials containing Ultima Gold scintillation liquid for counting. The level of radioactivity was corrected for protein content. (n = 7/6 biological replicates per group and 3 technical replicates.)

### RT Q-PCR

C2C12 cells were incubated for 24 h with chow or HFD CM containing 2% FBS. After 24 h, medium was aspirated and cells were washed two times with PBS. TRIzol reagent (Life Technologies) was added and plates were frozen until RNA isolation. Reverse transcription was performed on 300 ng total RNA (iScript cDNA Synthesis Kit, Bio-Rad). Gene products were determined by quantitative real-time PCR (CFX384 Touch Real-Time PCR Detection System, Bio-Rad) using iQ SYBR Green Supermix (Bio-Rad) for the following genes: *Murf1, Atrogin1, mtor, 4ebp1, Il6, Cxcl1, Cxcl2, Bnip3, Lc3b, Iκbα, Redd1, Mcp1, Icam1* (**Table 1**). The relative quantification was calculated using the ΔΔCt method, with *Rplp0* as the housekeeping gene. Values were normalized to the chow condition. (n = 6/7 biological replicates per group and 3 technical replicates).

**Table 1:** Primers used for quantitative real-time PCR.

Gene	Forward primer (5’ ➔ 3’)	Reverse primer (5’ ➔ 3’)
*Rplp0*	GGACCCGAGAAGACCTCCTT	GCACATCACTCAGAATTTCAATGG
*Mtor*	TCCTGCGCAAGATGCTCATC	TGTGCTCCAGCTCTGTCAGGA
*4ebp1*	CGGAAGATAAGCGGGCAG	CAGTGTCTGCCTGGTATGAG
*Murf1*	CTTCCTCTCAAGTGCCAAGCA	GTGTTCTAAGTCCAGAGTAAAGTAGTCCAT
*Redd1*	TCGGCGCTTCACTACTGACC	CCTAACACCCACCCCATTCC
*Atrogin1*	CAGCAGCTGAATAGCATCCAGAT	TCTGCATGATGTTCAGTTGTAAGC
*Bnip3*	AGGTTTTCCTTCCATCTCTGTTACTG	TGTGTGAACAGAAGTCAGATCCAAA
*Lc3b*	GAGCAGCACCCCACCAAGAT	CGTGGTCAGGCACCAGGAA
*Cxcl1*	TCGTCTTTCATATTGTATGGTCAACACG	TGCCCTACCAACTAGACACAAAATGTC
*Cxcl2*	CCCTGGTTCAGAAAATCATCCAAA	TTTGGTTCTTCCGTTGAGGGAC
*Il6*	ACAAGTCGGAGGCTTAATTACACAT	AATCAGAATTGCCATTGCACAA
*Iκβα*	GCTACCCGAGAGCGAGGAT	GCCTCCAAACACACAGTCATCA
*Mcp1*	CCTGCTGTTCACAGTTGCC	ATTGGGATCATCTTGCTGGT

### RNA Sequencing

RNA sequencing was performed at Maastricht University. Sequencing libraries were prepared from 500 ng of total RNA using the NEXTFLEX Rapid Directional RNA-Seq kit v2.0 with NEXTFLEX Poly(a) beads and unique dual indices. The libraries were sequenced in 100 bp paired-end on an SP flow cell (200 cycles) of the Illumina NovaSeq 6000, at an average of 72.4 million clusters passing filter per samples (min:51.4 M, max:88.7 M). The obtained raw data was first trimmed using fastp and the remaining reads were mapped to the Ensembl mouse genome (release 100) using STAR (version 2.7.3a) (18) and transcripts quantified using RSEM (v.1.3.1) (19) using default settings. The resulting raw read counts were normalized and processed using the R package DESeq2 (20) using default settings. Lowly expressed genes, defined as gene with more than 75% of the samples of any given condition not sequenced (i.e. 0 reads), were not considered in downstream analyses. An absolute fold change of >1.2 and an adjusted p-value of <0.05 was required for a gene to be considered significantly differentially expressed between conditions. Abnormal samples were filtered out according to the result of principal component analysis. The raw data is available in the European Nucleotide Archive (ENA) under the accession identifier ERP130473.

### Functional Enrichment Analysis

The Kyoto Encyclopedia of Genes and Genomes (KEGG) database and Gene Ontology (GO) category database were applied for functional annotation of differentially expressed genes. Enrichment analysis of KEGG and GO categories was performed using ConsensusPathDB (mpg.de) (20-05-2021).

### Protein Identification Using LC-MS/MS

Proteomics analysis was performed in HFD and chow CM. After acetone precipitation, a total of 10 µg protein in 25 µL 50 mM ammonium bicarbonate (ABC) with 5 M urea was used. 2.5 µL of dithiothreitol (DTT) solution (20 mM final) was added and incubated at room temperature for 45 min. The proteins were alkylated by adding 3 µL of indole-3-acetic acid (IAA) solution (40 mM final). The reaction took place at room temperature for 45 min in the dark. The alkylation was stopped by adding 5 µL of DTT solution (to consume any unreacted IAA) and incubated at room temperature for 45 min. For the digestion, 1 µg trypsin/LysC was added to the protein and incubated at 37°C for 2 h. 100 µL of 50 mM ABC was added to lower the urea concentration, and samples were further incubated at 37°C for 18 h. The digestion mix was centrifuged at 2,500 g for 5 min and the supernatant was collected for liquid chromatography with tandem mass spectrometry (LC-MS/MS) analysis. A nanoflow high-performance liquid chromatography instrument (Dionex UltiMate 3000) was coupled online to a Q Exactive (Thermo Scientific) with a nano-electrospray Flex ion source (Proxeon). 5 µL of the digest was loaded onto a C18-reversed phase column (Thermo Scientific, Acclaim PepMap C18 column, 75 µm inner diameter × 15 cm, 2 µm particle size). The peptides were separated with a 90 min linear gradient of 4-68% buffer B (80% acetonitrile and 0.08% formic acid) at a flow rate of 300 nL/min. MS data was acquired using a data-dependent top-10 method, dynamically choosing the most abundant precursor ions from the survey scan (280-1400 m/z) in positive ion mode. Survey scans were acquired at a resolution of 70,000 and a maximum injection time of 120 ms. Dynamic exclusion duration was 30 s. Isolation of precursors was performed with a 1.8 m/z window and a maximum injection time of 200 ms. Resolution for HCD spectra was set to 30,000 and the Normalized Collision Energy was 32 eV. The under-fill ratio was defined as 1.0%. The instrument was run with peptide recognition mode enabled, but with exclusion of singly charged and charge states of more than five.

### Database Search and Quantification

The MS data was searched using the Proteome Discoverer 2.2 Sequest HT search engine (Thermo Scientific), against the UniProt mouse database. The false discovery rate was set to 0.01 for proteins and peptides, which had to have a minimum length of 6 amino acids. The precursor mass tolerance was set at 10 ppm and the fragment tolerance at 0.02 Da. One miss-cleavage was tolerated, and oxidation of methionine was set as a dynamic modification. Carbamidomethylation of cysteines was set as fixed modification. Label-free quantification was conducted using the Minora Feature Detector node in the processing step and the Feature Mapper node combined with the Precursor Ions Quantifier node in the consensus step with default settings within Proteome Discoverer 2.2. The mass spectrometry proteomics data has been deposited to the ProteomeXchange Consortium *via* the PRIDE partner repository with the dataset identifier PXD027332.

### Statistical Analyses

Data is expressed as means ± SE and depicted as fold change compared to baseline of chow animals in bar charts. Mouse 13 (HFD) was not used for perfusion and liver slicing, due to a logistical problem. Due to an infection in the CM, mouse 1 (chow), and mouse 15 (HFD) were excluded from all analyses. The D’Agostino-Pearson test confirmed normal distribution of the data. Statistical analyses were performed by using unpaired Student’s t-tests or two-way ANOVA. (Note that in **Supplementary Figure 1**, IGF-1 is added to show the effect of a stimulus other than insulin, but has not been included in the statistical analysis.) Pearson’s correlation coefficient was used to evaluate the strength and direction of association between variables. All statistical analyses were performed with GraphPad Prism version 5.00 for Windows (GraphPad Software, Inc., San Diego, CA, USA). Statistical significance was set at p<0.05.

## Results

### Body Mass, Epididymal Fat Mass and Liver Fat

**Figure 1:**
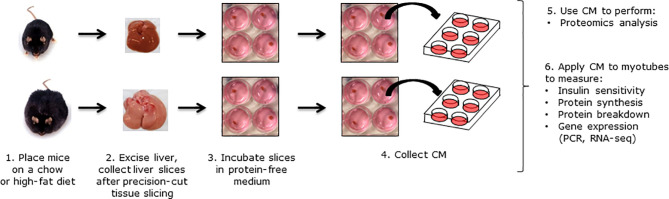
Overview of the study design. C57BL/6J mice were fed a chow or high-fat diet (HFD) for 12 weeks. Livers were perfused, excised and sliced into precision-cut liver slices. Liver slices were incubated in protein-free medium for 24 h, and CM was collected for experiments.

**Figure 2:**
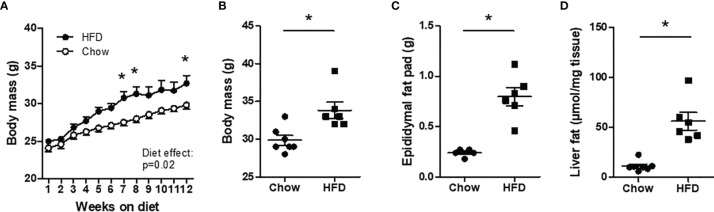
Body mass characteristics in chow and HFD mice. C57BL/6J mice were fed a chow (n=7) or high-fat diet (HFD) (n=6) for 12 weeks. **(A, B)** Body mass. **(C)** Epididymal fat mass. **(D)** Liver fat. *P < 0.05 *versus* chow.

An overview of the experimental design is depicted in **Figure 1**. Body mass was not different at the start of the diet and increased progressively in both groups over the duration of the experiment (**Figure 2A**). The increase in body mass was more pronounced in HFD mice compared with chow mice (diet effect p=0.02; **Figure 2A**). After 12 weeks, there was a modest but significant difference in body weight between both groups (28.9 ± 0.6 and 33.5 ± 1.2 g in the chow and HFD group, respectively; p<0.05) (**Figure 2B**). Epididymal fat mass was 3.3-fold higher in HFD mice compared with chow mice (**Figure 2C**). Liver TAG was 11.24 µmol/mg liver tissue (corresponding to 1.0% liver fat) in chow mice and 56.17 µmol/mg liver tissue (corresponding to 5.0% liver fat) in HFD mice (**Figure 2D**).

### Glucose Uptake in L6-GLUT4myc Myotubes After Exposure to CM

**Figure 3:**
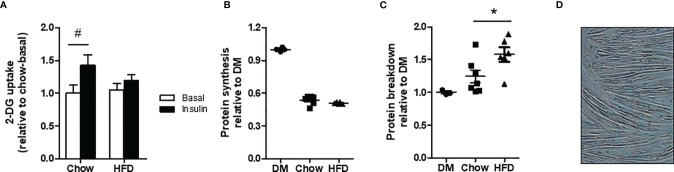
Insulin sensitivity and protein turnover in differentiated myotubes incubated with DM, chow CM or HFD CM. **(A)** 2-deoxyglucose (DG) uptake in L6-GLUT4myc myotubes without (basal) or with 10 nM insulin. n=7/6 biological replicates per group and 3 technical replicates. **(B)** Protein synthesis: L-[^14^C(U)]-phenylalanine incorporation in C2C12 cells incubated with chow or HFD CM. n=7/6 biological replicates and 4 technical replicates. **(C)** Protein breakdown L-[^14^C(U)]-phenylalanine release from pre-loaded C2C12 cells. n=7/6 biological replicates and 3 technical replicates. **(D)** C2C12 myotubes differentiated for 5 days.^#^P < 0.05 *versus* basal, *P < 0.05 *versus* chow.

Glucose uptake in L6-GLUT4myc myotubes was not different between chow and HFD CM in the basal state. Insulin-stimulated glucose uptake increased by 46% in myotubes exposed to chow CM but only by 18% in myotubes exposed to HFD CM (**Figure 3A**), demonstrating impaired insulin sensitivity in the myotubes incubated with HFD CM (interaction effect p<0.05).

### Protein Synthesis and Breakdown in C2C12 Myotubes After Exposure to CM

C2C12 cells differentiated for 3 days and incubated with DM show an increase in protein synthesis rates when stimulated with insulin and IGF-1 of 57% and 52%, respectively, compared with the unstimulated condition. Furthermore, C2C12 cells incubated with chow CM and HFD CM show an increase in protein synthesis rates of 62% and 67%, respectively, in the insulin-stimulated condition, compared with no stimulation. No differences in protein synthesis rates were found between chow and HFD CM in the basal condition, or in the stimulated condition (**Supplementary Figure 1**). Also after 5 days of differentiation, protein synthesis rates did not differ between chow and HFD CM (**Figure 3B**; p=0.11). Protein breakdown rates were increased by 27% in HFD CM compared with chow CM (**Figure 3C**). As shown in **Figure 3D**, cells were fully differentiated into myotubes by day 5.

### Correlative Analysis

**Figure 4:**
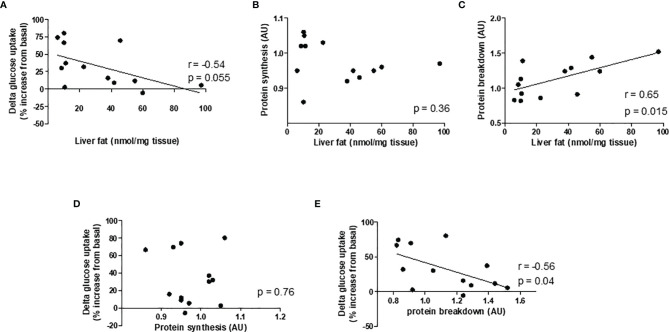
Pearson's correlation graphs to evaluate the strength and direction of asssociation between variables. Correlation between **(A)** liver fat and delta glucose uptake, **(B)** liver fat and protein synthesis, **(C)** liver fat and protein breakdown, **(D)** protein synthesis and delta glucose uptake, and **(E)** protein breakdown and delta glucose uptake. n = 7 chow and 6 HFD.

Given our hypothesis that hepatic steatosis causes muscle insulin resistance and muscle protein loss, and to investigate a possible link between insulin resistance and muscle protein synthesis and breakdown rates, correlative analysis was performed. Liver TAG tended to be negatively correlated with delta glucose uptake, which was calculated by the difference between basal glucose and insulin-stimulated glucose uptake (r= -0.54, p=0.055; **Figure 4A**). Liver TAG was positively correlated with protein breakdown rates (r=0.65, p=0.015), but did not correlate with protein synthesis rates (**Figures 4B, C**). Protein breakdown rates also correlated negatively with delta glucose uptake (r= -0.56, p=0.04); there was no correlation between protein synthesis rates and delta glucose uptake (**Figures 4D, E**).

### Gene Expression in C2C12 Myotubes After Exposure to CM

**Figure 5:**
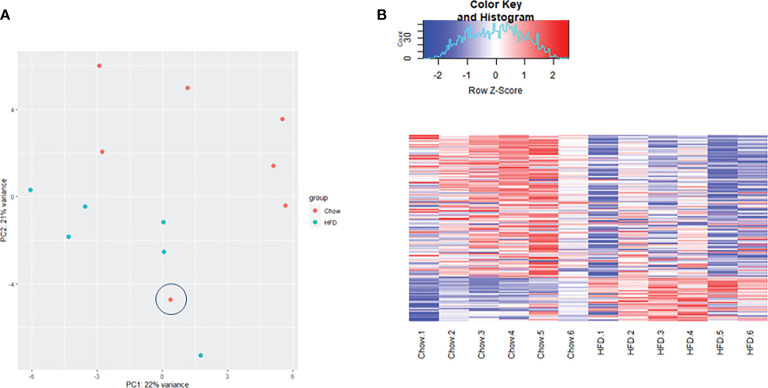
Gene sequencing data analysis. **(A)** Principal component analaysis (PCA) demonstrating gene clustering by group (diet) with the exception of one outlier(encircled). **(B)** Comparative heat map analysis of all 197 differentially expressed genes in C2C12 cells incubated with HFD CM (n=6) compared with C2C12 cells incubated with chow CM (n=6) after exclusion of the outlier. Red indicates higher expression, and blue indicates lower expression. Expression values are normalized per row, using Z-score transform.

**Figure 6:**
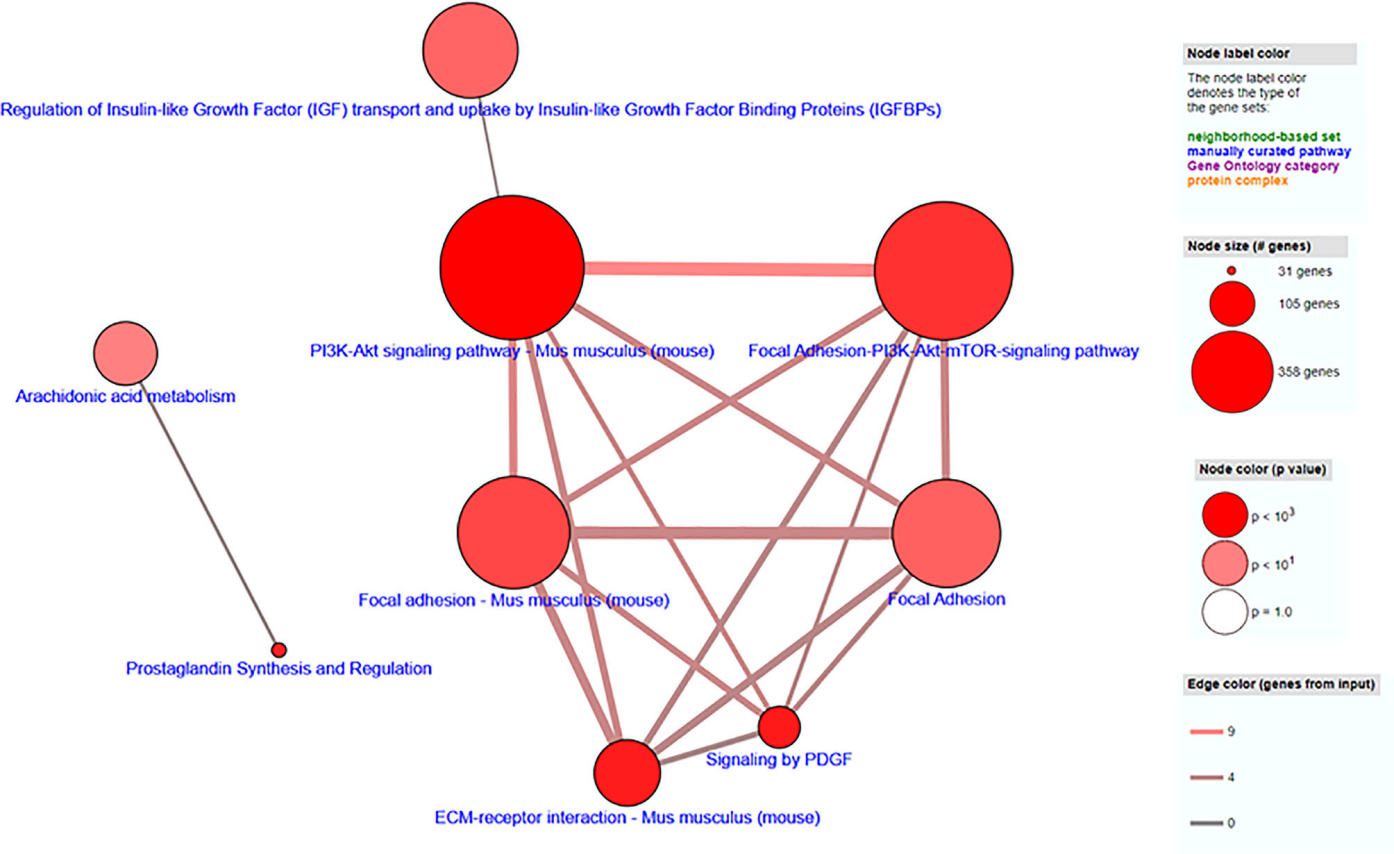
Visualization of pathway enrichment analysis of differentially expressed genes, indicating links between pathways. Each node represents a separate concept whose member list size (i.e., number of genes contained) and P-value are encoded as node size and node color, respectively. Two nodes are connected by an edge if they share more than 1 member. The edge width reflects the relative overlap between the nodes, while the edge color encodes the number of shared genes.

Transcriptome profiling was performed on C2C12 cells incubated with either HFD or chow CM. Principal component analysis indicated that both groups were well separated into distinct clusters, with the exception of one outlier (see **Figure 5A**; scores for principal component 1 *versus* 2 shown). In total, 12,585 expressed genes were detected, of which only 21 genes were differentially expressed between groups (adjusted p < 0.05). After exclusion of the outlier, 197 genes were found to be differentially expressed, of which 46 (23.4%) were upregulated and 151 (76.6%) were downregulated (a heat map is presented in **Figure 5B**). These 197 genes were used for further analysis. To gain insight into the biological function of the differentially expressed genes, pathway enrichment analysis was performed. Pathways defined by WikiPathways, Reactome, and KEGG were included in the analysis. Only pathways with a minimum overlap of 4 genes between our dataset and a respective pathway were considered. There were 12 significantly enriched pathways, including ‘PI3K-Akt signaling pathway’, ‘Signaling by PDGF’, ‘ECM-receptor interaction’, ‘Prostaglandin Synthesis and Regulation’, ‘focal adhesion-PI3K-Akt-mTOR-signaling pathway’, ‘spinal cord injury’, ‘focal adhesion - Mus musculus’, ‘focal adhesion’, ‘Regulation of Insulin-like Growth Factor (IGF) transport and uptake by Insulin-like Growth Factor Binding Proteins’, and ‘arachidonic acid metabolism’. The pathways identified by pathway analysis are ranked by p-value and presented in **Table 2**. Links between relevant pathways are visualized in **Figure 6**. Exploration of the list of differentially expressed genes showed that multiple genes play a role in multiple enriched pathways, confirming the close link between pathways (**Table 3**). Gene ontology enrichment analysis was also performed, and showed that the differentially expressed genes were mostly involved in ‘system development’, ‘anatomical structure development’, ‘multicellular organism development’, ‘regulation of anatomical structure morphogenesis’, ‘animal organ development’, ‘vasculature development’, ‘anatomical structure morphogenesis’, ‘cardiovascular system development’, ‘blood vessel development’, and ‘cellular developmental process’ (**Table 4**).

**Table 2:** Top enriched pathways-based sets of differentially expressed genes.

Pathway name	Pathway source	Set size	Candidates contained	p-value	q-value
PI3K-Akt signaling pathway - Mus musculus (mouse)	KEGG	358	13 (3.6%)	6.3 e-05	0.004
Signaling by PDGF	Reactome	52	5 (9.6%)	0.0002	0.004
ECM-receptor interaction - Mus musculus (mouse)	KEGG	83	6 (7.2%)	0.0002	0.004
Prostaglandin Synthesis and Regulation	WikiPathways	31	4 (12.9%)	0.0003	0.004
Human papillomavirus infection - Mus musculus (mouse)	KEGG	370	12 (3.3%)	0.0003	0.004
Focal Adhesion-PI3K-Akt-mTOR-signaling pathway	WikiPathways	322	11 (3.4%)	0.0004	0.004
Spinal Cord Injury	WikiPathways	101	6 (5.9%)	0.0005	0.005
Amoebiasis - Mus musculus (mouse)	KEGG	106	6 (5.7%)	0.0006	0.005
Focal adhesion - Mus musculus (mouse)	KEGG	199	8 (4.0%)	0.0009	0.006
Focal Adhesion	WikiPathways	182	7 (3.8%)	0.0024	0.016
Regulation of Insulin-like Growth Factor (IGF) transport and uptake by Insulin-like Growth Factor Binding Proteins (IGFBPs)	Reactome	141	6 (4.3%)	0.0029	0.018
Arachidonic acid metabolism	Reactome	79	4 (5.1%)	0.0083	0.046

**Table 3:** Differentially expressed genes in eight of the enriched pathways.

	Change in HFD CM compared with chow CM	p-value, not adjusted	p-value, adjusted for multiple testing	PI3K-Akt signaling pathway - Mus musculus	Signaling by PDGF	ECM-receptor interaction	Prostaglandin Synthesis and Regulation	Focal Adhesion-PI3K-Akt-mTOR-signaling pathway	Focal adhesion - Mus musculus	Regulation of IGF transport and uptake by IGFBPs	Arachidonic acid metabolism
Arachidonate 5-lipoxygenase	↓	0.000	0.000								x
CD44 antigen	↓	0.000	0.000			x					
Platelet-derived growth factor, D polypeptide	↓	0.000	0.000	x	x			x	x		
Aldo-keto reductase family 1, member C14	↓	0.000	0.000								x
Granzyme E	↓	0.000	0.000							x	
Apolipoprotein L 9b	↓	0.000	0.000							x	
Insulin-like growth factor binding protein 2	↓	0.000	0.001							x	
Laminin, beta 3	↓	0.000	0.001	x		x		x	x		
Myosin, light chain 12B, regulatory	↓	0.000	0.006						x		
Thrombospondin 2	↓	0.000	0.006	x	x	x		x	x		
Integrin alpha 4	↓	0.000	0.009	x		x		x	x		
Annexin A1	↓	0.000	0.009				x				
Angiopoietin 4	↓	0.000	0.025								
Thrombospondin 1	↓	0.000	0.025	x	x	x		x	x		
cAMP responsive element binding protein 5	↓	0.000	0.027	x				x			
Platelet-derived growth factor, C polypeptide	↓	0.000	0.027	x	x			x	x		
S100 calcium binding protein A6 (calcyclin)	↓	0.000	0.030				x				
Collagen, type IV, alpha 5	↓	0.001	0.035	x	x	x			x		
Cysteine rich protein 61	↓	0.001	0.038							x	
Chordin-like 1	↓	0.001	0.040							x	
Cellular communication network factor 1	↓	0.001	0.044								
protein phosphatase 2, regulatory subunit B, beta	↓	0.001	0.037	x				x			
Prostaglandin-endoperoxide synthase 2	↑	0.000	0.000				x				x
Serum/glucocorticoid regulated kinase 1	↑	0.000	0.000	x				x			
6-phosphofructo-2-kinase/fructose-2,6biphosphatase 3	↑	0.000	0.001								
Phospholipase A2, group IVA (cytosolic, ca-dependent)	↑	0.000	0.014				x				x
Interleukin 6	↑	0.000	0.023	x						x	
Interleukin 4 receptor, alpha	↑	0.001	0.037	x				x			

**Table 4:** Top 20 enriched gene ontology-based sets of differentially expressed genes.

Gene ontology term	Set size	Candidates contained	p-value	q-value
GO:0048731 system development	4787	88 (1.8%)	2.72e-13	8.41e-11
GO:0048856 anatomical structure development	5856	99 (1.7%)	4.74e-13	5.2e-11
GO:0007275 multicellular organism development	5339	93 (1.7%)	8.59e-13	5.2e-11
GO:0022603 regulation of anatomical structure morphogenesis	1065	33 (3.1%)	4.07e-10	2.15e-07
GO:0048513 animal organ development	3602	66 (1.8%)	2.07e-09	3.2e-07
GO:0001944 vasculature development	746	25 (3.4%)	1.52e-08	3.84e-06
GO:0009653 anatomical structure morphogenesis	2701	54 (2.0%)	6.19e-09	2.5e-07
GO:0072358 cardiovascular system development	760	25 (3.3%)	2.19e-08	3.84e-06
GO:0001568 blood vessel development	715	24 (3.4%)	2.94e-08	1.82e-06
GO:0048869 cellular developmental process	4445	73 (1.6%)	2.35e-08	6.2e-07
GO:0071495 cellular response to endogenous stimulus	1289	34 (2.6%)	1.27e-08	1.31e-06
GO:0009888 tissue development	1952	43 (2.2%)	2.3e-08	1.77e-06
GO:0071495 cellular response to endogenous stimulus	1289	34 (2.6%)	2.57e-08	1.59e-06
GO:0070887 cellular response to chemical stimulus	2962	55 (1.9%)	4.51e-08	2.32e-06
GO:0009719 response to endogenous stimulus	1600	38 (2.4%)	2.56e-08	6.2e-07
GO:0050793 regulation of developmental process	2722	51 (1.9%)	1.55e-07	6e-06
GO:2000026 regulation of multicellular organismal development	2141	44 (2.1%)	1.11e-07	1.47e-05
GO:0010035 response to inorganic substance	583	21 (3.6%)	7.13e-08	3.15e-06
GO:0072359 circulatory system development	1143	29 (2.5%)	4.01e-07	4.22e-05
GO:0030154 cell differentiation	4246	67 (1.6%)	6.14e-07	1.96e-05

### PCR Analysis

**Figure 7:**
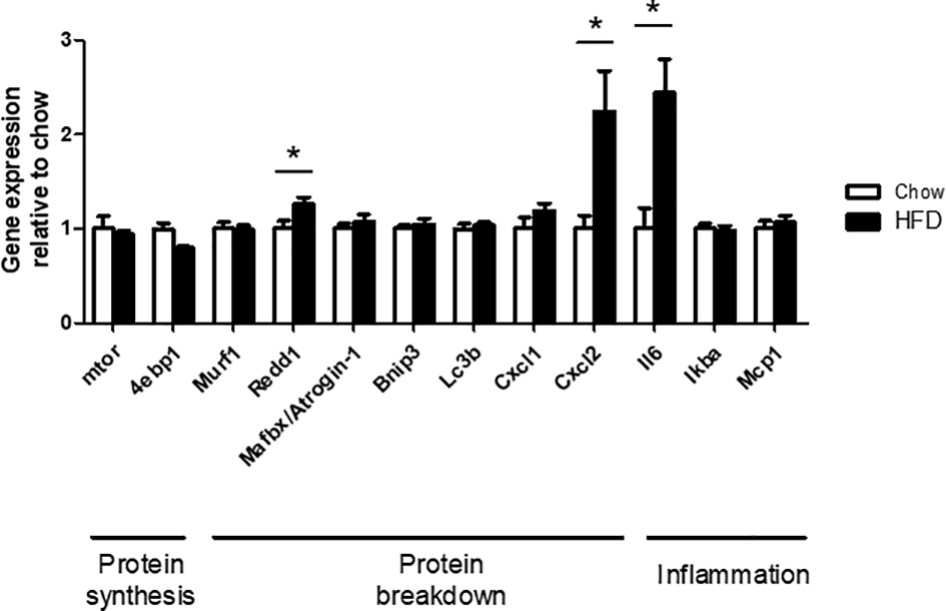
PCR analysis of genes with functions related to protein synthesis, protein breakdown, and inflammation. Gene expression in differentiated C2C12 cells, treated with chow or HFD CM, and expressed relative to chow. n=7/6 biological replicates and 3 technical replicates. *P < 0.05 *versus* chow.

Expression of genes with important roles in protein synthesis, protein breakdown, and inflammation were examined *via* PCR. There were no differences in the expression of *mtor, 4ebp1, Murf1, Atrogin1, Bnip3, Lc3b, Cxcl1, Iκbα*, and *Mcp1* in C2C12 cells treated with HFD CM compared with chow CM. In contrast, *Redd1, Cxcl2, *and *Il6* expression were increased in C2C12 cells treated with HFD CM compared with chow CM, by 2.4-fold, 2.2-fold, and 1.3-fold, respectively (**Figure 7**).

### Proteomics Analysis in Chow and HFD CM

Using LC-MS/MS, 1,045 unique proteins were found to be present in the CM and using SignalP 5.0 and Deeploc 154 proteins were identified as secreted proteins. Specifically, SignalP 5.0 detected the presence of a signal peptide sequence in 152 proteins whereas Deeploc recognized 90 secreted proteins by predicting an extracellular location. Of these 154 proteins, 32 proteins were found to be differentially expressed in HFD CM, with a fold change >1.2 and an adjusted p-value <0.05 compared with chow CM. Of these proteins, 30 proteins were downregulated and 2 were upregulated. To gain better insight into the biological function of the differentially expressed proteins, pathway analysis was performed. Pathways defined by WikiPathways, Reactome, and KEGG were included in the analysis. Only pathways with a minimum overlap of 4 proteins between our dataset and a respective pathway were considered. All enriched pathways were ‘regulation of Insulin-like Growth Factor (IGF) transport and uptake by Insulin-like Growth Factor Binding Proteins (IGFBPs)’, ‘Post-translational protein phosphorylation’, ‘Phase I - Functionalization of compounds’, ‘biological oxidations’, ‘Protein processing in endoplasmic reticulum - Mus musculus’, ‘Drug metabolism - other enzymes - Mus musculus’, ‘Platelet degranulation’, ‘Response to elevated platelet cytosolic Ca^2+^’, ‘platelet activation, signaling and aggregation’, ‘post-translational modification’, ‘metabolism of proteins’, ‘hemostasis’, ‘neutrophil degranulation’, and ‘immune system’. The top 10 enriched gene ontology-based sets include ‘endoplasmic reticulum lumen’, ‘extracellular space’, ‘endoplasmic reticulum’, ‘endoplasmic reticulum part’, ‘endomembrane system’, ‘protein folding’, ‘endoplasmic reticulum chaperone complex’, ‘protein disulfide isomerase activity’, ‘intramolecular oxidoreductase activity, transposing S-S bonds’, and ‘cell redox homeostasis’.

## Discussion

In the past decade, an increasing amount of evidence suggests a strong relationship between NAFLD and sarcopenia. The direction of the relationship, however, is not clear and the majority of studies suggest that muscle loss increases the risk of developing NAFLD (9). In this study, we evaluated whether secretion products from fatty mouse livers affect protein synthesis and breakdown rates in cultured myotubes. C57BL/6J mice were fed standard chow or a HFD for 12 weeks, livers were excised and sliced, and secretion products from cultured liver slices were collected and placed on differentiated C2C12 or L6-GLUT4myc cells. There were no differences in protein synthesis rates, but there was an increased insulin resistance and increased protein breakdown in myotubes incubated with HFD CM compared with chow CM. Furthermore, pathway analysis of C2C12 gene expression showed that multiple pathways related to anatomical structure and function were enriched. These findings support our hypothesis that the secretome of a fatty liver contributes to the development of muscle loss in individuals with NAFLD.

Mechanisms underlying the development of sarcopenia in the context of inter-organ crosstalk are heavily understudied. It has only been in the past 15 years that studies have slowly started to emerge, suggesting a role for adipose tissue in the development of muscle wasting. An early study in 2007 showed that C2C12 cells exposed to saturated fatty acids (palmitate) and unsaturated fatty acids (oleate) increased protein degradation by 25% and 18%, respectively, and this degradation was ameliorated by adiponectin (21). It was also found that C2C12 cells treated with CM from differentiated human adipocytes not only displayed impaired insulin signaling, but also significantly reduced expression of myogenin (22), a protein that is required for the recruitment of the transcription initiation machinery to muscle-specific genes (23). In 2015, Pellegrinelli et al. placed the secretome of human obese adipocytes on differentiated human primary satellite cells and demonstrated a decreased expression of contractile proteins in myotubes, consequently inducing atrophy (24). The investigators also established that adipocytes from visceral adipose tissue depots were more potent than adipocytes from subcutaneous adipose tissue depots in provoking deleterious effects in muscle cells (24). Very recently, Okun et al. were the first to show a causal link between altered liver function and decreased muscle mass (25). Specifically, the authors found that the expression of alanine aminotransferases was increased in the liver in mice with obesity and diabetes, as well as in humans with type 2 diabetes, and hepatocyte-selective silencing of alanine aminotransferase enzymes in mice with obesity and diabetes retarded hyperglycemia and reversed skeletal muscle atrophy through restoration of skeletal muscle protein synthesis (25).

In our study, we found increased protein breakdown rates in C2C12 cells incubated with HFD CM, and we observed a positive correlation between the amount of liver fat and protein breakdown rates in C2C12 cells. This is a remarkable observation, as it supports our hypothesis that hepatic fat accumulation is responsible for the increase in muscle protein breakdown. Interestingly, myotubes incubated with HFD CM also developed insulin resistance, and there was a significant correlation between protein breakdown in C2C12 cells and delta glucose uptake in L6-GLUT4myc cells, indicating that those CM samples that resulted in a high level of insulin resistance, also induced high levels of protein breakdown. It may be possible that the liver secretome is directly responsible for the decrease in insulin sensitivity as well as for the increase in protein breakdown in C2C12 cells. Alternatively, it is possible that the liver secretome decreases protein breakdown *via* a decrease in insulin sensitivity. Also *in vivo* studies previously reported a positive correlation between insulin sensitivity and lean body mass (26), and multiple papers reported that insulin resistance accelerates muscle protein degradation (27, 28) and inhibits protein synthesis (29). Interesting findings regarding the link between insulin sensitivity and muscle mass were also reported by Mitch et al. who created insulin-deficient rats by injection of streptozotocin. They found that muscle protein degradation was increased by 75% (30), whereas treatment of these rats with insulin for ≥24 h reversed muscle proteolysis and returned mRNAs to control levels (31), suggesting a role for insulin in the maintenance of muscle mass. Similar results were found in subjects with type 1 diabetes, in which insulin therapy inhibited protein breakdown (32). Thus, it seems that insulin and insulin sensitivity play an important role in the maintenance of muscle mass.

Through RNA sequencing, 197 genes were found to be significantly changed in C2C12 cells incubated with HFD CM, compared with chow CM. Using pathway analysis, the top enriched pathway-based set was found to be ‘PI3-Akt signaling pathway’. This is an interesting observation, as the PI3-Akt signaling pathway is the major insulin-sensitive pathway resulting in glucose uptake. In line with this finding, we previously found that the secretome of a fatty liver indeed induced insulin resistance in muscle cells (6), and insulin resistance is assumed to play a major role in the development of muscle atrophy, as discussed earlier. The second pathway-based set that was found to be enriched was ‘signaling by PDGF’, which is also a remarkable finding. Platelet‐derived growth factors (PDGFs) are a family of growth factors expressed in skeletal muscle, and receptors for these proteins play important roles in muscle growth and remodeling (33). Specifically, PDGF signaling is required for fiber hypertrophy, extracellular matrix (ECM) production, and angiogenesis that occurs during muscle growth (33). The third pathway-based set that was enriched was ‘ECM-receptor interaction - Mus musculus’. The skeletal muscle ECM plays an important role in muscle fiber force transmission, maintenance, and repair, and the ‘ECM-receptor interaction pathway’ includes interactions within the ECM that lead to control of cellular activities such as adhesion, migration, differentiation, proliferation, and apoptosis. In case of injury or disease, the ECM adapts dramatically resulting in clinical manifestations and altered muscle function (34). Three other enriched pathways that are of specific interest are related to focal adhesion (**Figure 6**). Focal adhesions are large multi-protein structures that form mechanical links between intracellular actin bundles and the ECM, and proteins associate and disassociate with it continually as signals are transmitted to other parts of the cell (35). Interestingly, PI3K/Akt and mTOR were indicated as two pathways affected by changes in focal adhesion; pathways that are well-known to play key roles in the regulation of insulin sensitivity and muscle growth (36, 37). ‘Prostaglandin Synthesis and Regulation’ and ‘Arachidonic acid metabolism’ are also relevant pathways that were enriched. Prostaglandins are mainly synthesized from arachidonic acid and are known to be major regulators of skeletal muscle protein turnover and exercise training adaptations (38, 39). Altogether, our results suggest that HFD CM leads toward a dysregulation of a set of genes with important roles in the maintenance of muscle mass and health.

Exploration of the list of differentially expressed genes showed that multiple genes play a role in multiple enriched pathways, confirming the close link between pathways (**Table 4**). Several of those genes are well-known players involved in the regulation of muscle morphology and muscle function, including thrombospondin-1 (Thbs1) and thrombospondin-2 (Thbs2) (40–43), annexin A1 (44–47), S100 calcium-binding protein A6 (calcyclin) (48), prostaglandin-endoperoxide synthase 2 (Ptgs2 or COX2) (49–51), and phospholipase A2, group IVA (cytosolic, calcium-dependent) (cPLA2) (52, 53). Previously, studies have tried to identify a set of genes that are differentially expressed with different causes of muscle loss, but this has appeared challenging. Genome-wide expression profiling analysis was used to identify the molecular changes that occur in two mouse models of muscle atrophy: hindlimb casting and Achilles tendon laceration. Interestingly though, both disuse models of skeletal muscle atrophy induced very distinct protein degradation profiles (54). Other studies, however, successfully identified a set of genes that play a role in the development of muscle wasting, which include *Atrogin1/Mafbx* and *Murf1* (55). Interestingly, we could not detect any changes in *Atrogin1/Mafbx* and *Murf1*, as measured by RNA sequencing and PCR analysis. In agreement with this, it has previously been described that in some conditions of muscle wasting (e.g. sarcopenia), there are discrepancies between studies, showing upregulation, downregulation, or no alteration in Atrogin-1/MAFbx and MuRF-1 expression (56). Especially in studies with COPD patients, a patient group that may be relatively similar to NAFLD patients, 3 of the 4 studies found no change in MuRF-1. Also in rats, in which a relatively modest dose of IL-6 was infused locally, muscle atrophy was induced in the absence of any changes in Atrogin-1/MAFbx and MuRF-1 (57). Also other important modulators of muscle protein synthesis and breakdown were not changed in our study, including *mtor, 4ebp1, Bnip3, and Iκβ*α. PCR analysis did, however, (as well as RNA sequencing) reveal an increased expression of *Il6* and *Redd1* of 2.4 and 1.3-fold, respectively. IL-6 is a cytokine with both pro-inflammatory and anti-inflammatory properties, and with a variety of functions related to metabolism (58). Chronically increased IL-6 levels have been linked to mitochondrial dysfunction (59), and sarcopenia (60). Furthermore, IL-6 has been suggested to contribute to the development of type 2 diabetes (61), and intervention studies identified IL-6 as a key regulator of muscle mass during cachexia (57, 62). REDD1 is a known inhibitor of the Akt/mTOR signaling pathway and studies found a role for REDD1 in the regulation of cell growth, mitochondrial function, oxidative stress, and apoptosis, leading to tissue damage (63). Notably, even though PCR and gene sequencing analysis are of great use to provide an overview of the pathways that are affected by a particular intervention, they do not give any information on post-translational modifications and phosphorylation states of individual proteins, while these latter aspects are also important to take into account.

In our study, we used PCLS as model. De Graaf et al. previously showed that PCLS represent a mini-model of the liver and contain all cells of the tissue in their natural environment, leaving intercellular and cell-matrix interactions intact (17). PCLS are therefore highly appropriate for studying multicellular processes, and represent a better physiological model to study inter-organ crosstalk between liver and skeletal muscle, compared with isolated hepatocytes. Pro-inflammatory cytokines have previously been linked to the development of muscle wasting in obese patients (64). To investigate whether protein secretion from PCLS may be responsible for the change in gene expression in C2C12 cells incubated with HFD CM compared with chow CM, proteomics analysis was performed in chow and HFD CM. It was previously found that diet-induced steatosis alters the liver transcriptome and proteome profile in mice and in humans (65–68), resulting in a different hepatokine secretion profile compared to lean livers (6). In our current study, we found that 32 proteins were differentially expressed in HFD CM compared with chow CM. Of these proteins, 30 proteins were downregulated and 2 were upregulated (**Supplementary Table 1**), indicating a pronounced downregulation. However, we did not see a difference between the chow and HFD CM with respect to the absolute amount of proteins secreted, which indicates that this is not explained by an overall downregulation of liver function and secretion. Also, data is normalized for total protein content per sample. Nevertheless, pathway analysis indicated that the differentially expressed proteins had important functions with respect to Insulin-like Growth Factor (IGF) transport and uptake, thereby showing a link with insulin sensitivity and protein turnover. Other interesting pathways include ‘post-translational protein phosphorylation’, ‘protein processing in endoplasmic reticulum’, ‘post-translational modification’, and ‘metabolism of proteins’. These findings suggest that HFD CM not only affects gene expression in skeletal muscle, but also affects post-translational processes, including protein folding, protein secretion, and protein phosphorylation. In line with this, gene ontology analysis found indeed that processes in the endoplasmic reticulum and the extracellular space were strongly enriched.

The concept of inter-organ crosstalk between liver and skeletal muscle in the development of muscle atrophy is relatively new. In the current study, we found evidence that secretion products from a fatty liver negatively affect protein breakdown. Nevertheless, despite evidence to support a role for the liver in the development of muscle atrophy, it cannot be excluded that low muscle mass also affects liver health. Similar to the liver, skeletal muscle is an endocrine organ that secretes myokines, and these may affect liver function *via* inter-organ crosstalk. It has previously been shown that muscle-derived IL-6 plays a role in triggering glucose output from the liver during exercise in humans (69). In addition, the myokine irisin has been suggested to suppress the progression of hepatic fibrosis by regulating hepatic stellate cell activation, proliferation, migration, contractility, and hepatic stellate cell-mediated production of inflammatory cytokines (70). Furthermore, blocking myostatin was shown to increase muscle mass, ameliorate liver insulin resistance, and decrease hepatic steatosis in HFD mice (71). Thus, although direct evidence is still scarce, it is plausible that decreased muscle health, oxidative stress, and inflammation may lead to an increased secretion of harmful myokines, which may contribute to NAFLD progression. Further detailed mechanistic studies investigating the link between NAFLD and sarcopenia are needed.

There are some other limitations in our study. One limitation is that only male mice were included. It is known that pre-menopausal female individuals are less prone to develop hepatic steatosis and metabolic problems upon weight gain, compared with males. This is likely due to different hormones, less visceral fat, more gluteofemoral fat, less fat spillover, and thus less ectopic fat accumulation (72, 73). This difference between males and females has also been shown in mice (74, 75). To investigate the link between early stage NAFLD and the development of muscle atrophy, we wanted to use a model in which we could induce liver steatosis and metabolic problems in a short amount of time; hence, we chose male mice. Nevertheless, it would be of great interest to look specifically at sex differences in relation to adiposity, depot differences, liver fat, and muscle mass in future research, particularly in humans.

It is also important to note that, apart from proteins, other products may contribute to the effects observed. It is now well-appreciated that tissues secrete proteins, lipids, metabolites, and small non-coding RNAs which impact local functions in an autocrine/paracrine manner, and also influence biological processes in distant tissues. The effect of these secretory products should be topic of future research.

In conclusion, this study provides evidence that secretion products from a fatty liver lead to profound changes in skeletal muscle gene expression, and increased muscle protein breakdown rates. The study supports the hypothesis that hepatic steatosis contributes to the development of muscle atrophy in individuals with NAFLD.

## Data Availability Statement

The datasets presented in this study can be found in online repositories. The data presented in the study are deposited in the PRIDE Archive repository at https://www.ebi.ac.uk/pride/archive/, accession number PXD027332, and in the European Nucleotide Archive repository at https://www.ebi.ac.uk/ena, accession number ERP130473.

## Ethics Statement

The animal study was reviewed and approved by the Animal Ethics Committee of Maastricht University.

## Author Contributions

LL, EB, and RM designed the study. KP and RM performed experiments. FS, SO, PO, and RL provided essential materials and/or technical expertise. KP, MA, FC, and RM analyzed the data. KP, RL, SR, EB, LL, and RM discussed and interpreted the findings. KP and RM wrote the manuscript. All authors contributed to the article and approved the submitted version.

## Funding

RM was supported by a “Marie Skłodowska Curie individual fellowship” by the European Commission (H2020-MSCA-IF).

## Conflict of Interest

The authors declare that the research was conducted in the absence of any commercial or financial relationships that could be construed as a potential conflict of interest.

## Publisher’s Note

All claims expressed in this article are solely those of the authors and do not necessarily represent those of their affiliated organizations, or those of the publisher, the editors and the reviewers. Any product that may be evaluated in this article, or claim that may be made by its manufacturer, is not guaranteed or endorsed by the publisher.

## References

[1] Younossi ZM Koenig AB Abdelatif D Fazel Y Henry L Wymer M. Global Epidemiology of Nonalcoholic Fatty Liver Disease-Meta-Analytic Assessment of Prevalence, Incidence, and Outcomes. Hepatology (2016) 64(1):73–84. doi: 10.1002/hep.28431 26707365

[2] Browning JD Szczepaniak LS Dobbins R Nuremberg P Horton JD Cohen JC. Prevalence of Hepatic Steatosis in an Urban Population in the United States: Impact of Ethnicity. Hepatology (2004) 40(6):1387–95. doi: 10.1002/hep.20466 15565570

[3] Silverman JF O’Brien KF Long S Leggett N Khazanie PG Pories WJ. Liver Pathology in Morbidly Obese Patients With and Without Diabetes. Am J Gastroenterol (1990) 85(10):1349–55. 2220728

[4] Schwenger KJP Fischer SE Jackson TD Okrainec A Allard JP. Non-Alcoholic Fatty Liver Disease in Morbidly Obese Individuals Undergoing Bariatric Surgery: Prevalence and Effect of the Pre-Bariatric Very Low Calorie Diet. Obes Surg (2018) 28(4):1109–16. doi: 10.1007/s11695-017-2980-3 29098545

[5] Bellentani S Saccoccio G Masutti F Croce LS Brandi G Sasso F. Prevalence of and Risk Factors for Hepatic Steatosis in Northern Italy. Ann Intern Med (2000) 132(2):112–7. doi: 10.7326/0003-4819-132-2-200001180-00004 10644271

[6] Meex RC Hoy AJ Morris A Brown RD Lo JC Burke M. Fetuin B Is a Secreted Hepatocyte Factor Linking Steatosis to Impaired Glucose Metabolism. Cell Metab (2015) 22(6):1078–89. doi: 10.1016/j.cmet.2015.09.023 26603189

[7] El Sherif O Dhaliwal A Newsome PN Armstrong MJ. Sarcopenia in Nonalcoholic Fatty Liver Disease: New Challenges for Clinical Practice. Expert Rev Gastroenterol Hepatol (2020) 14(3):197–205. doi: 10.1080/17474124.2020.1731303 32064966

[8] von Haehling S Morley JE Anker SD. An Overview of Sarcopenia: Facts and Numbers on Prevalence and Clinical Impact. J Cachexia Sarcopenia Muscle (2010) 1(2):129–33. doi: 10.1007/s13539-010-0014-2 PMC3060646 21475695

[9] Cai C Song X Chen Y Chen X Yu C. Relationship Between Relative Skeletal Muscle Mass and Nonalcoholic Fatty Liver Disease: A Systematic Review and Meta-Analysis. Hepatol Int (2020) 14(1):115–26. doi: 10.1007/s12072-019-09964-1 PMC6994447 31290072

[10] Petta S Ciminnisi S Di Marco V Cabibi D Camma C Licata A. Sarcopenia Is Associated With Severe Liver Fibrosis in Patients With Non-Alcoholic Fatty Liver Disease. Aliment Pharmacol Ther (2017) 45(4):510–8. doi: 10.1111/apt.13889 28028821

[11] Koo BK Kim D Joo SK Kim JH Chang MS Kim BG. Sarcopenia Is an Independent Risk Factor for Non-Alcoholic Steatohepatitis and Significant Fibrosis. J Hepatol (2017) 66(1):123–31. doi: 10.1016/j.jhep.2016.08.019 27599824

[12] Carias S Castellanos AL Vilchez V Nair R Dela Cruz AC Watkins J. Nonalcoholic Steatohepatitis Is Strongly Associated With Sarcopenic Obesity in Patients With Cirrhosis Undergoing Liver Transplant Evaluation. J Gastroenterol Hepatol (2016) 31(3):628–33. doi: 10.1111/jgh.13166 PMC6615558 26399838

[13] Kim G Kang SH Kim MY Baik SK. Prognostic Value of Sarcopenia in Patients With Liver Cirrhosis: A Systematic Review and Meta-Analysis. PLoS One (2017) 12(10):e0186990. doi: 10.1371/journal.pone.0186990 29065187 PMC5655454

[14] Hong HC Hwang SY Choi HY Yoo HJ Seo JA Kim SG. Relationship Between Sarcopenia and Nonalcoholic Fatty Liver Disease: The Korean Sarcopenic Obesity Study. Hepatology (2014) 59(5):1772–8. doi: 10.1002/hep.26716 23996808

[15] Moon JS Yoon JS Won KC Lee HW. The Role of Skeletal Muscle in Development of Nonalcoholic Fatty Liver Disease. Diabetes Metab J (2013) 37(4):278–85. doi: 10.4093/dmj.2013.37.4.278 PMC3753493 23991406

[16] Bergerson JT Lee JG Furlan A Sourianarayanane A Fetzer DT Tevar AD. Liver Transplantation Arrests and Reverses Muscle Wasting. Clin Transplant (2015) 29(3):216–21. doi: 10.1111/ctr.12506 25557648

[17] de Graaf IA Olinga P de Jager MH Merema MT de Kanter R van de Kerkhof EG. Preparation and Incubation of Precision-Cut Liver and Intestinal Slices for Application in Drug Metabolism and Toxicity Studies. Nat Protoc (2010) 5(9):1540–51. doi: 10.1038/nprot.2010.111 20725069

[18] Dobin A Davis CA Schlesinger F Drenkow J Zaleski C Jha S. STAR: Ultrafast Universal RNA-Seq Aligner. Bioinformatics (2013) 29(1):15–21. doi: 10.1093/bioinformatics/bts635 23104886 PMC3530905

[19] Li B Dewey CN. RSEM: Accurate Transcript Quantification From RNA-Seq Data With or Without a Reference Genome. BMC Bioinf (2011) 12:323. doi: 10.1186/1471-2105-12-323 PMC3163565 21816040

[20] Love MI Huber W Anders S. Moderated Estimation of Fold Change and Dispersion for RNA-Seq Data With Deseq2. Genome Biol (2014) 15(12):550. doi: 10.1186/s13059-014-0550-8 25516281 PMC4302049

[21] Zhou Q Du J Hu Z Walsh K Wang XH. Evidence for Adipose-Muscle Cross Talk: Opposing Regulation of Muscle Proteolysis by Adiponectin and Fatty Acids. Endocrinology (2007) 148(12):5696–705. doi: 10.1210/en.2007-0183 17761767

[22] Sell H Eckardt K Taube A Tews D Gurgui M Van Echten-Deckert G. Skeletal Muscle Insulin Resistance Induced by Adipocyte-Conditioned Medium: Underlying Mechanisms and Reversibility. Am J Physiol Endocrinol Metab (2008) 294(6):E1070–7. doi: 10.1152/ajpendo.00529.2007 18364460

[23] Adhikari A Kim W Davie J. Myogenin Is Required for Assembly of the Transcription Machinery on Muscle Genes During Skeletal Muscle Differentiation. PloS One (2021) 16(1):e0245618. doi: 10.1371/journal.pone.0245618 33465133 PMC7815108

[24] Pellegrinelli V Rouault C Rodriguez-Cuenca S Albert V Edom-Vovard F Vidal-Puig A. Human Adipocytes Induce Inflammation and Atrophy in Muscle Cells During Obesity. Diabetes (2015) 64(9):3121–34. doi: 10.2337/db14-0796 25695947

[25] Okun JG Rusu PM Chan AY Wu Y Yap YW Sharkie T. Liver Alanine Catabolism Promotes Skeletal Muscle Atrophy and Hyperglycaemia in Type 2 Diabetes. Nat Metab (2021) 3(3):394–409. doi: 10.1038/s42255-021-00369-9 33758419

[26] Unni US Ramakrishnan G Raj T Kishore RP Thomas T Vaz M. Muscle Mass and Functional Correlates of Insulin Sensitivity in Lean Young Indian Men. Eur J Clin Nutr (2009) 63(10):1206–12. doi: 10.1038/ejcn.2009.32 19471290

[27] Wang X Hu Z Hu J Du J Mitch WE. Insulin Resistance Accelerates Muscle Protein Degradation: Activation of the Ubiquitin-Proteasome Pathway by Defects in Muscle Cell Signaling. Endocrinology (2006) 147(9):4160–8. doi: 10.1210/en.2006-0251 16777975

[28] Price SR Gooch JL Donaldson SK Roberts-Wilson TK. Muscle Atrophy in Chronic Kidney Disease Results From Abnormalities in Insulin Signaling. J Ren Nutr (2010) 20(5 Suppl):S24–8. doi: 10.1053/j.jrn.2010.05.007 PMC2937009 20797566

[29] Stephens FB Chee C Wall BT Murton AJ Shannon CE van Loon LJ. Lipid-Induced Insulin Resistance Is Associated With an Impaired Skeletal Muscle Protein Synthetic Response to Amino Acid Ingestion in Healthy Young Men. Diabetes (2015) 64(5):1615–20. doi: 10.2337/db14-0961 25524913

[30] Price SR Bailey JL Wang X Jurkovitz C England BK Ding X. Muscle Wasting in Insulinopenic Rats Results From Activation of the ATP-Dependent, Ubiquitin-Proteasome Proteolytic Pathway by a Mechanism Including Gene Transcription. J Clin Invest (1996) 98(8):1703–8. doi: 10.1172/JCI118968 PMC507607 8878419

[31] Mitch WE Bailey JL Wang X Jurkovitz C Newby D Price SR. Evaluation of Signals Activating Ubiquitin-Proteasome Proteolysis in a Model of Muscle Wasting. Am J Physiol (1999) 276(5):C1132–8. doi: 10.1152/ajpcell.1999.276.5.C1132 10329962

[32] Nair KS Ford GC Ekberg K Fernqvist-Forbes E Wahren J. Protein Dynamics in Whole Body and in Splanchnic and Leg Tissues in Type I Diabetic Patients. J Clin Invest (1995) 95(6):2926–37. doi: 10.1172/JCI118000 PMC295981 7769135

[33] Sugg KB Korn MA Sarver DC Markworth JF Mendias CL. Inhibition of Platelet-Derived Growth Factor Signaling Prevents Muscle Fiber Growth During Skeletal Muscle Hypertrophy. FEBS Lett (2017) 591(5):801–9. doi: 10.1002/1873-3468.12571 PMC5352504 28129672

[34] Gillies AR Lieber RL. Structure and Function of the Skeletal Muscle Extracellular Matrix. Muscle Nerve (2011) 44(3):318–31. doi: 10.1002/mus.22094 PMC3177172 21949456

[35] Wozniak MA Modzelewska K Kwong L Keely PJ. Focal Adhesion Regulation of Cell Behavior. Biochim Biophys Acta (2004) 1692(2-3):103–19. doi: 10.1016/j.bbamcr.2004.04.007 15246682

[36] Latres E Amini AR Amini AA Griffiths J Martin FJ Wei Y. Insulin-Like Growth Factor-1 (IGF-1) Inversely Regulates Atrophy-Induced Genes *via* the Phosphatidylinositol 3-Kinase/Akt/Mammalian Target of Rapamycin (PI3K/Akt/Mtor) Pathway. J Biol Chem (2005) 280(4):2737–44. doi: 10.1074/jbc.M407517200 15550386

[37] Schmitz-Peiffer C. Signalling Aspects of Insulin Resistance in Skeletal Muscle: Mechanisms Induced by Lipid Oversupply. Cell Signal (2000) 12(9-10):583–94. doi: 10.1016/S0898-6568(00)00110-8 11080610

[38] Liu SZ Jemiolo B Lavin KM Lester BE Trappe SW Trappe TA. Prostaglandin E2/Cyclooxygenase Pathway in Human Skeletal Muscle: Influence of Muscle Fiber Type and Age. J Appl Physiol (1985) (2016) 120(5):546–51. doi: 10.1152/japplphysiol.00396.2015 PMC4773645 26607246

[39] Naruse M Fountain WA Claiborne A Chambers TL Jones AM Stroh AM. Influence of Low-Dose Aspirin, Resistance Exercise, and Sex on Human Skeletal Muscle PGE2/COX Pathway Activity. Physiol Rep (2021) 9(5):e14790. doi: 10.14814/phy2.14790 33661544 PMC7931802

[40] Zhang K Li M Yin L Fu G Liu Z. Role of Thrombospondin1 and Thrombospondin2 in Cardiovascular Diseases (Review). Int J Mol Med (2020) 45(5):1275–93. doi: 10.3892/ijmm.2020.4507 PMC7138268 32323748

[41] Yamashiro Y Thang BQ Shin SJ Lino CA Nakamura T Kim J. Role of Thrombospondin-1 in Mechanotransduction and Development of Thoracic Aortic Aneurysm in Mouse and Humans. Circ Res (2018) 123(6):660–72. doi: 10.1161/CIRCRESAHA.118.313105 PMC6211815 30355232

[42] Watkins SC Lynch GW Kane LP Slayter HS. Thrombospondin Expression in Traumatized Skeletal Muscle. Correlation of Appearance With Post-Trauma Regeneration. Cell Tissue Res (1990) 261(1):73–84. doi: 10.1007/BF00329440 2200612

[43] Kyriakides TR Maclauchlan S. The Role of Thrombospondins in Wound Healing, Ischemia, and the Foreign Body Reaction. J Cell Commun Signal (2009) 3(3-4):215–25. doi: 10.1007/s12079-009-0077-z PMC2778594 19844806

[44] Seidel S Neymeyer H Kahl T Roschel T Mutig K Flower R. Annexin A1 Modulates Macula Densa Function by Inhibiting Cyclooxygenase 2. Am J Physiol Renal Physiol (2012) 303(6):F845–54. doi: 10.1152/ajprenal.00704.2011 22791338

[45] Yoon JH Kim D Jang JH Ghim J Park S Song P. Proteomic Analysis of the Palmitate-Induced Myotube Secretome Reveals Involvement of the Annexin A1-Formyl Peptide Receptor 2 (FPR2) Pathway in Insulin Resistance. Mol Cell Proteomics (2015) 14(4):882–92. doi: 10.1074/mcp.M114.039651 PMC4390267 25616869

[46] Goto-Inoue N Tamura K Motai F Ito M Miyata K Manabe Y. A Fragmented Form of Annexin A1 Is Secreted From C2C12 Myotubes by Electric Pulse-Induced Contraction. Mol Cell Biochem (2016) 411(1-2):173–80. doi: 10.1007/s11010-015-2579-8 26458561

[47] Bizzarro V Fontanella B Franceschelli S Pirozzi M Christian H Parente L. Role of Annexin A1 in Mouse Myoblast Cell Differentiation. J Cell Physiol (2010) 224(3):757–65. doi: 10.1002/jcp.22178 20578244

[48] Jurewicz E Robaszkiewicz K Moraczewska J Filipek A. Binding of S100A6 to Actin and the Actin-Tropomyosin Complex. Sci Rep (2020) 10(1):12824. doi: 10.1038/s41598-020-69752-y 32733033 PMC7393103

[49] Chen H Qian Z Zhang S Tang J Fang L Jiang F. Silencing COX-2 Blocks PDK1/TRAF4-Induced AKT Activation to Inhibit Fibrogenesis During Skeletal Muscle Atrophy. Redox Biol (2021) 38:101774. doi: 10.1016/j.redox.2020.101774 33152664 PMC7645269

[50] Mantovani G Maccio A Madeddu C Serpe R Antoni G Massa E. Phase II Nonrandomized Study of the Efficacy and Safety of COX-2 Inhibitor Celecoxib on Patients With Cancer Cachexia. J Mol Med (Berl) (2010) 88(1):85–92. doi: 10.1007/s00109-009-0547-z 19802504

[51] Lai V George J Richey L Kim HJ Cannon T Shores C. Results of a Pilot Study of the Effects of Celecoxib on Cancer Cachexia in Patients With Cancer of the Head, Neck, and Gastrointestinal Tract. Head Neck (2008) 30(1):67–74. doi: 10.1002/hed.20662 17615567

[52] Haq S Kilter H Michael A Tao J O’Leary E Sun XM. Deletion of Cytosolic Phospholipase A2 Promotes Striated Muscle Growth. Nat Med (2003) 9(7):944–51. doi: 10.1038/nm891 12808451

[53] Pharaoh G Brown JL Sataranatarajan K Kneis P Bian J Ranjit R. Targeting Cpla2 Derived Lipid Hydroperoxides as a Potential Intervention for Sarcopenia. Sci Rep (2020) 10(1):13968. doi: 10.1038/s41598-020-70792-7 32811851 PMC7435184

[54] Bialek P Morris C Parkington J St Andre M Owens J Yaworsky P. Distinct Protein Degradation Profiles Are Induced by Different Disuse Models of Skeletal Muscle Atrophy. Physiol Genomics (2011) 43(19):1075–86. doi: 10.1152/physiolgenomics.00247.2010 PMC3217324 21791639

[55] Lecker SH Jagoe RT Gilbert A Gomes M Baracos V Bailey J. Multiple Types of Skeletal Muscle Atrophy Involve a Common Program of Changes in Gene Expression. FASEB J (2004) 18(1):39–51. doi: 10.1096/fj.03-0610com 14718385

[56] Rom O Reznick AZ. The Role of E3 Ubiquitin-Ligases Murf-1 and Mafbx in Loss of Skeletal Muscle Mass. Free Radic Biol Med (2016) 98:218–30. doi: 10.1016/j.freeradbiomed.2015.12.031 26738803

[57] Haddad F Zaldivar F Cooper DM Adams GR. IL-6-Induced Skeletal Muscle Atrophy. J Appl Physiol (1985) (2005) 98(3):911–7. doi: 10.1152/japplphysiol.01026.2004 15542570

[58] Mauer J Denson JL Bruning JC. Versatile Functions for IL-6 in Metabolism and Cancer. Trends Immunol (2015) 36(2):92–101. doi: 10.1016/j.it.2014.12.008 25616716

[59] Abid H Ryan ZC Delmotte P Sieck GC Lanza IR. Extramyocellular Interleukin-6 Influences Skeletal Muscle Mitochondrial Physiology Through Canonical JAK/STAT Signaling Pathways. FASEB J (2020) 34(11):14458–72. doi: 10.1096/fj.202000965RR 32885495

[60] Rong YD Bian AL Hu HY Ma Y Zhou XZ. Study on Relationship Between Elderly Sarcopenia and Inflammatory Cytokine IL-6, Anti-Inflammatory Cytokine IL-10. BMC Geriatr (2018) 18(1):308. doi: 10.1186/s12877-018-1007-9 30541467 PMC6292155

[61] Akbari M Hassan-Zadeh V. IL-6 Signalling Pathways and the Development of Type 2 Diabetes. Inflammopharmacology (2018) 26(3):685–98. doi: 10.1007/s10787-018-0458-0 29508109

[62] Carson JA Baltgalvis KA. Interleukin 6 as a Key Regulator of Muscle Mass During Cachexia. Exerc Sport Sci Rev (2010) 38(4):168–76. doi: 10.1097/JES.0b013e3181f44f11 PMC3065300 20871233

[63] Britto FA Dumas K Giorgetti-Peraldi S Ollendorff V Favier FB. Is REDD1 a Metabolic Double Agent? Lessons From Physiology and Pathology. Am J Physiol Cell Physiol (2020) 319(5):C807–24. doi: 10.1152/ajpcell.00340.2020 32877205

[64] Levine ME Crimmins EM. The Impact of Insulin Resistance and Inflammation on the Association Between Sarcopenic Obesity and Physical Functioning. Obes (Silver Spring) (2012) 20(10):2101–6. doi: 10.1038/oby.2012.20 PMC3527629 22310233

[65] Schmid GM Converset V Walter N Sennitt MV Leung KY Byers H. Effect of High-Fat Diet on the Expression of Proteins in Muscle, Adipose Tissues, and Liver of C57BL/6 Mice. Proteomics (2004) 4(8):2270–82. doi: 10.1002/pmic.200300810 15274121

[66] Kirpich IA Gobejishvili LN Bon Homme M Waigel S Cave M Arteel G. Integrated Hepatic Transcriptome and Proteome Analysis of Mice With High-Fat Diet-Induced Nonalcoholic Fatty Liver Disease. J Nutr Biochem (2011) 22(1):38–45. doi: 10.1016/j.jnutbio.2009.11.009 20303728 PMC3860361

[67] Greco D Kotronen A Westerbacka J Puig O Arkkila P Kiviluoto T. Gene Expression in Human NAFLD. Am J Physiol Gastrointest Liver Physiol (2008) 294(5):G1281–7. doi: 10.1152/ajpgi.00074.2008 18388185

[68] Younossi ZM Baranova A Ziegler K Del Giacco L Schlauch K Born TL. A Genomic and Proteomic Study of the Spectrum of Nonalcoholic Fatty Liver Disease. Hepatology (2005) 42(3):665–74. doi: 10.1002/hep.20838 16116632

[69] Febbraio MA Hiscock N Sacchetti M Fischer CP Pedersen BK. Interleukin-6 Is a Novel Factor Mediating Glucose Homeostasis During Skeletal Muscle Contraction. Diabetes (2004) 53(7):1643–8. doi: 10.2337/diabetes.53.7.1643 15220185

[70] Dong HN Park SY Le CT Choi DH Cho EH. Irisin Regulates the Functions of Hepatic Stellate Cells. Endocrinol Metab (Seoul) (2020) 35(3):647–55. doi: 10.3803/EnM.2020.658 PMC7520590 32981307

[71] Wilkes JJ Lloyd DJ Gekakis N. Loss-of-Function Mutation in Myostatin Reduces Tumor Necrosis Factor Alpha Production and Protects Liver Against Obesity-Induced Insulin Resistance. Diabetes (2009) 58(5):1133–43. doi: 10.2337/db08-0245 PMC2671051 19208906

[72] Ayonrinde OT Olynyk JK Beilin LJ Mori TA Pennell CE de Klerk N. Gender-Specific Differences in Adipose Distribution and Adipocytokines Influence Adolescent Nonalcoholic Fatty Liver Disease. Hepatology (2011) 53(3):800–9. doi: 10.1002/hep.24097 21374659

[73] Lonardo A Nascimbeni F Ballestri S Fairweather D Win S Than TA. Sex Differences in Nonalcoholic Fatty Liver Disease: State of the Art and Identification of Research Gaps. Hepatology (2019) 70(4):1457–69. doi: 10.1002/hep.30626 PMC6766425 30924946

[74] Jacobs SAH Gart E Vreeken D Franx BAA Wekking L Verweij VGM. Sex-Specific Differences in Fat Storage, Development of Non-Alcoholic Fatty Liver Disease and Brain Structure in Juvenile HFD-Induced Obese Ldlr-/-.Leiden Mice. Nutrients (2019) 11(8):1861. doi: 10.3390/nu11081861 PMC6723313 31405127

[75] Arisqueta L Navarro-Imaz H Labiano I Rueda Y Fresnedo O. High-Fat Diet Overfeeding Promotes Nondetrimental Liver Steatosis in Female Mice. Am J Physiol Gastrointest Liver Physiol (2018) 315(5):G772–80. doi: 10.1152/ajpgi.00022.2018 30095299

