# Supplementary figures and images for: Hepatic Steatosis Contributes to the Development of Muscle Atrophy via Inter-Organ Crosstalk
Source: Front Endocrinol (Lausanne). 2021 Oct 11;12:733625. doi: 10.3389/fendo.2021.733625 (PMC8542925; doi:10.3389/fendo.2021.733625)

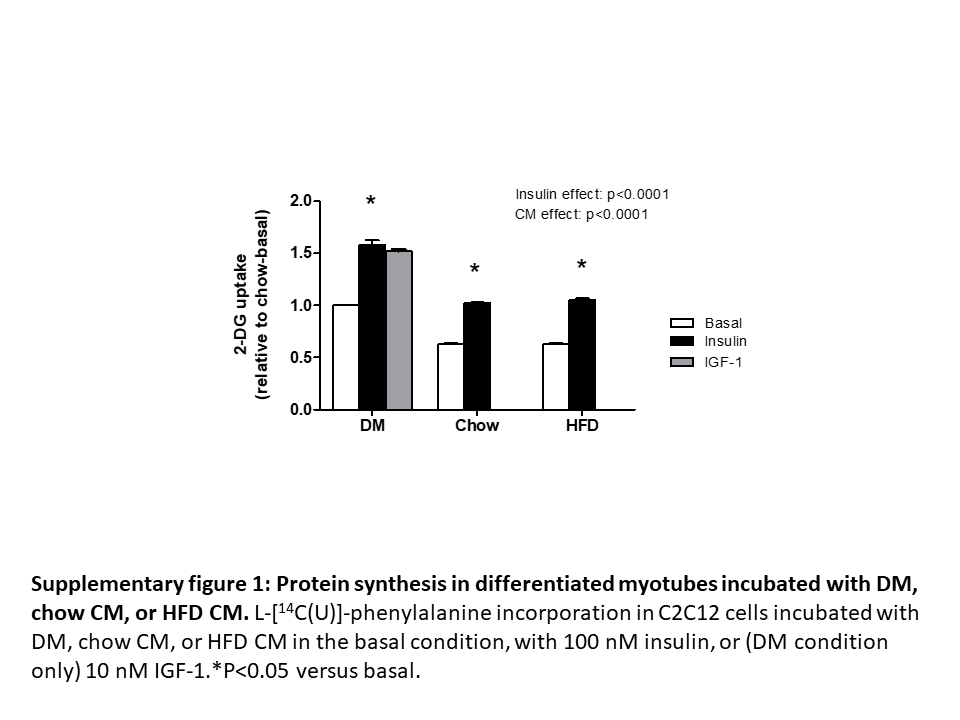

Supplement: Supplementary file 1 [file Image_1.tif]
