# Supplementary material for: Hepatic Steatosis Contributes to the Development of Muscle Atrophy via Inter-Organ Crosstalk
Source: Front Endocrinol (Lausanne). 2021 Oct 11;12:733625. doi: 10.3389/fendo.2021.733625 (PMC8542925; doi:10.3389/fendo.2021.733625)
Supplement: Supplementary file 2 [file Table_1.docx]

Supplementary table 1. Differentially expressed proteins between chow and HFD CM.

| **Protein** | **Accession**  **number** | **Fold change** | **q-value** |
| --- | --- | --- | --- |
| Serine protease inhibitor A3K | P07759 | -2.2 | 0.00 |
| Endoplasmic reticulum resident protein 44 | Q9D1Q6 | -2.1 | 0.01 |
| Acylcarnitine hydrolase | Q91WG0 | -3.4 | 0.01 |
| Pyrethroid hydrolase Ces2a | Q8QZR3-1 | -3.7 | 0.01 |
| Cysteine-rich with EGF-like domain protein 2 | Q9CYA0 | -6.6 | 0.01 |
| Calreticulin | P14211 | -1.6 | 0.01 |
| Serotransferrin | Q921I1 | -2.0 | 0.02 |
| peptidyl-prolyl cis-trans isomerase B | P24369 | -1.7 | 0.02 |
| Protein disulfide-isomerase A4 | P08003 | -1.5 | 0.02 |
| Carboxylesterase 3B | Q8VCU1-1 | -2.1 | 0.02 |
| Carboxylesterase 3A | Q63880 | -1.9 | 0.02 |
| Peroxiredoxin-4 | O08807 | -1.7 | 0.03 |
| 78 kDa glucose-regulated protein | P20029 | -1.3 | 0.03 |
| Plasminogen | P20918 | -1.6 | 0.03 |
| Calumenin | O35887 | -3.3 | 0.03 |
| Murinoglobulin-1 | P28665 | -2.4 | 0.03 |
| Carboxypeptidase Q | Q9WVJ3 | -1.7 | 0.03 |
| Carboxylesterase 1E | Q64176 | -1.6 | 0.03 |
| Protein disulfide-isomerase A6 | Q922R8 | -2.2 | 0.03 |
| Isoform 2 of Glucosidase 2 subunit beta | O08795-2 | -1.9 | 0.04 |
| alpha-2-macroglobulin receptor-associated protein | P55302 | -1.7 | 0.03 |
| Carboxylesterase 1D | Q8VCT4 | -1.8 | 0.03 |
| H-2 class I histocompatibility antigen, D-D alpha chain | P01900 | 1.7 | 0.04 |
| Pyrethroid hydrolase Ces2e | Q8BK48 | -1.7 | 0.04 |
| Alpha-1-antitrypsin 1-4 | Q00897 | -1.7 | 0.04 |
| Inhibitor of carbonic anhydrase | Q9DBD0 | -4.3 | 0.04 |
| Cadherin-2 | P15116 | 1.7 | 0.04 |
| endoplasmic reticulum aminopeptidase 1 | Q9EQH2 | -1.7 | 0.04 |
| Protein disulfide-isomerase A3 | P27773 | -1.3 | 0.04 |
| Antithrombin-III | P32261 | -1.5 | 0.04 |
| Liver carboxylesterase 1 | Q8VCC2 | -1.8 | 0.05 |
| Transthyretin | P07309 | -1.6 | 0.05 |
